# Traité Sur Les Maladies Du Foie

**Published:** 1837-01-01

**Authors:** 


### Traité sur les Maladies du Foie. Par *A. Bonnet*. Paris, 1836.

" All inflammations of the liver are secondary to a gastro-enteritis." Such is one of the propositions formally announced, and much insisted upon, in Broussais' pathology; and of this, as of almost all the others, we may safely assert that it is partly true and partly false. It is but fair to state that the great medical reformer of the nineteenth century has not, perhaps, carried his own dogmas so far, nor urged them with such unqualified assurance, as some of his followers have done, and are apt to do in the present day. With them, there appears to be scarcely any other abdominal disease but " gastro-enterite"—either as the primary affection, or as the all-important complication. Dyspepsia, according to their creed, is a gastro-enterite—so are gastrodynia and colic; jaundice and cholera are the same; every form of diarrhœa is invariably, we are told, dependent upon some inflammatory affection of the stomach and bowels; and the whole catalogue of various and varied fevers is forced to acknowledge the same genealogy. That Broussais has introduced some very important changes in the theory and in the practice of physic, that he has simplified our views of the true nature of many diseases, which before his time were ill understood and badly managed, that he has given greater precision to our principles of diagnosis, and that he has thereby inspired us with a more enlightened and a more secure confidence in our therapeutic arrangements, we are most willing and happy to confess. His is a great and a venerable name; and all who have traced the progress of the healing art since the beginning of the present century, will readily acknowledge that he has done more to its advancement than any other one of his cotemporaries. To justify in some degree his title to this distinguished praise, let us briefly consider the very topic with which we commenced these remarks—the connexion of hepatic inflammation with gastro-enteric disease. We have already expressed our dissent from the universal and unqualified admission of this doctrine; but that it is partially, nay, even very frequently true, we are bound to believe, from the results of an extensive and varied experience.

Numerous and most complicated are, it is well known, the sympathies which the intestinal tube holds with almost every part of the frame. A blow on the region of the stomach will double up the body, and bring on a state of almost fatal powerlessness and exhaustion; a mouthful or two of nauseous or oppressive food will change, in the course of a few minutes, the energy of vigorous and cheerful health into the puling discontent of a most uneasy restlessness. Think, then, of the miserable feelings of sea-sickness;—the utter prostration and helplessness, the total abandonment of spirit, the palsy of all the faculties, the swimming head, the sightless eyes, the tottering limbs: whence are all these, but from the universal sympathy with the vexed stomach? In short, nutrition being the fundamental function or process of life, and the intestinal canal being the laboratory where this process is effected, we are at once prepared to expect and rationally to account for the "*consensus* à pede usque ad calcem" of every part with the great centre of organic life. There was therefore not only much political shrewdness, but not a little physiological acumen also, in the Roman Senator's speech, when he appeased the discontented citizens, by telling them the fable of the mutiny of body's members against the seemingly indolent stomach:

"There was a time, when all the body's members
Rebelled against the belly; thus accused it—
That only like a gulf, it did remain
I' th' midst of the body, idle and inactive,
Still cupboarding the viand, never bearing
Like labour with the rest:
The belly answered;
True I receive the general food at first
Which you do live upon; and fit it is,
Because I am the storehouse and the shop
Of the whole body. But if you do remember,
I send it through the rivers of your blood,
Even to the court, the heart; to th' seat o' the brain;
And, through the cranks and offices of man,
The strongest nerves and small inferior veins
From me receive that natural competency,
Whereby they live."

In these words of our marvellous dramatist, we have an admirable physiological description of the intimate and necessary dependence of every part of the body on the state of the stomach. "The kingly-crowned head," "the vigilant eye," "the counsellor heart," "the arm our soldier," "our steed the leg," and "the tongue our trumpeter"—all must acknowledge the control of "the cormorant belly."

Need we wonder then, when we consider how all-powerful is the influence of the digestive organs not only on our physical, but also on our intellectual and moral capacities, that the ancients placed the seat of some of the passions, as anger, jealousy and melancholy, in the abdominal viscera.

But well known as all these considerations had been for many centuries, physicians seemed not to have suspected the extent of that influence which diseased states of the stomach and intestines may have on the features and progress of other maladies; and it was not until the publication of Broussais' Examen and Commentaires that the full importance of studying these relations was satisfactorily established. Dessault in France, and Dr. Hamilton and Mr. Abernethy in this country, contributed most essentially in preparing the way to the introduction of more correct etiological doctrines and of more judicious and successful therapeutic precepts. Indeed it is more than probable, that the attention of Broussais was especially directed to the investigation of the post-mortem appearances in the primæ viæ, from the beneficial changes which had been recently effected in the treatment of many diseases by these, his eminent predecessors, having so convincingly shewn that numerous forms of disease, external as well as internal, surgical as well as medical, are, if not actually caused, at least greatly modified and exasperated by a disordered condition of the stomach and bowels, and that more good may be derived from a regulated course of aperient medicines, than from the use of all the other members of the pharmaceutical catalogue.

This doctrine and this practice may have been pushed too far, and we really do believe that not a little empiricism has crept into British physic since Dr. Hamilton has published his excellent work on Purgatives. One of the errors, thus extensively propagated, is to be traced to the very general belief, that the almost constant disorder of the alimentary canal consisted merely in an accumulation of unhealthy bile and feculent matters, and that, therefore, the great therapeutic indication was to remove and expel these. Broussais has most satisfactorily shewn the fallacy and danger of such a doctrine in a numerous set of cases. He has proved that the train of symptoms so accurately described by Hamilton and Abernethy is often owing to a subacute inflammation of the mucous membrane of the alimentary tube, that the use of strong purgatives in these circumstances is injurious, and that a cure is to be effected by leeches, blisters, demulcents, and so forth.

These preliminary remarks may be useful to the reader before we call his attention to the contents of the book under review.

M. Bonnet is an ardent disciple of Broussais, and the critical reader may detect in almost every page of his work the tenets of the physiological school peering out. He starts with making the unqualified assertion, that every case of hepatitis is accompanied by inflammation of the peritoneal covering of the liver, and of the mucous surface of the stomach and duodenum. Even in those cases in which the liver becomes inflamed from penetrating wounds or from contusions, we are assured by him that the disease is always propagated not only to its peritoneal covering, but also to the alimentary canal; and, strange to say, he appeals to some cases, which in our opinion are rather favourable to the opposite side of the question. For example, the first case recorded was that of an officer who was wounded in fencing, by a sharp-pointed foil entering for several inches in the right hypochondriac region. Acute hepatitis came on; suppuration succeeded, and he died on the 12th day after the accident. The liver was found on dissection to be much enlarged and of a deep red colour. An abscess occupied the right lobe. "L'estomac et le duodenum etaient rouges égalément." Such were the only inflammatory appearances in the stomach and intestines, in a case which our prejudiced author holds up as "perfectly conclusive" of his doctrine! But the next case is even more unsatisfactory; it is taken from Portal's treatise on "Maladies du Foie." A man died on the eleventh day after an attack of most acute hepatitis, which had terminated in suppuration. The appearances discovered in the liver itself are minutely described; and all that we learn of the state of the alimentary tube is in these words: "la cavité de l'estomac etait un peu retrecie, et la partie qui etait unie au foie par les adherences des pseudo-ligaments parassait atteinte d'une legére inflammation, surtout dans la face interne."

So determined is M. Bonnet in maintaining his position, that he even calls in question the accuracy of those authors who have reported any cases of fatal hepatitis, in which no distinct traces of lesion were discoverable in the stomach and bowels. He rejects, for example, one or two such cases of M. Andral, and with no lack of presumption tells us that M. Andral must be mistaken; for the very existence of certain symptoms during the lives of the patients, as recorded in the reports, indicates an "irritation du tube alimentaire." Now before adducing other cases to shew the fallacy of M. Bonnet's assertions, we beg our readers' attention for a few minutes to this expression—"irritation du tube alimentaire." That this irritation, according to M. Bonnet himself, implies nothing more nor less than the presence of the usual symptoms of disordered stomach and bowels, such as nausea, anorexia, furred tongue, disagreeable taste in the mouth, tendency to vomiting, and irregularity of the alvine evacuations, is apparent from his own work; for in one part of it he alludes to the distinction between mere irritation and actual inflammation of the gastro-duodenal surface. Is it fair, therefore, or philosophical to ground his disbelief in the accuracy of M. Andral's cases on such flimsy quibbling? It is very evident that, in his indiscreet zeal for the doctrine which he advocates, he has had recourse to mere ambiguity of expression to bolster up its weak points.

No one ever dreamed of denying that the functions of the chylopoietic viscera are almost invariably, nay almost necessarily, disordered—or, if Bonnet prefers the language, that the gastro-enteric mucous surface is in a state of irritation—in diseases of the liver. It cannot in truth be otherwise; for all these organs are so intimately associated in the performance of a common process, that one of them cannot suffer, without involving more or less all the others. This most important law should never be lost sight of in investigating the nature, causes, and treatment of abdominal diseases; and we have already awarded to M. Broussais the praise of having illustrated its truth with great ability and success. What we find fault with in him and in his followers is, that they recognize only one form of complication in all cases of hepatitis—that form being, as a matter of course, gastro-enterite.

To justify our dissent from this doctrine, we may confidently appeal to the observation of every experienced physician; and were we in want of other data, reference might be made to a table of 25 cases of liver disease, which Dr. Stokes has inserted in the Cyclopædia of Practical Medicine, art., *Inflammation of the Liver*, with the view of illustrating this very topic. In one half of these cases, drawn partly from the practice of MM. Andral and Louis, and partly from his own, no traces of gastro-enteritis were to be found on dissection.

The circumstance, however, of this complication being present in the other half of the cases deserves to be noticed, as some valuable considerations are thence deducible, to regulate our conduct in a therapeutic point of view. The practice of immediately resorting to the use of acrid purgatives in liver disorders, without the state of the gastro-intestinal tube having been previously well investigated—a practice which has been especially prevalent among British physicians—is certainly very injudicious. The already inflamed mucous surface is thus exposed to the action of stimulants applied directly to it; the feverish symptoms are necessarily aggravated; the liver sympathises, and any diseased action, which is already established there, is increased.

Numerous are the cases, which twenty years ago would have been designated under the vague and very comprehensive term "bilious affection," and treated with active purgatives, not to mention a course of mercurials, but of which the true nature and primary cause is now ascertained to be a subacute inflammation of the upper portion of the intestinal tube, and which may be speedily relieved by a much milder and much safer practice. If Broussais had done nothing more for pathology than merely corrected the pernicious notions which were entertained of the nature of such cases as we have now alluded to, he would have deserved well of the medical profession.

We have said enough, we believe, on the frequency of gastro-enteritis being complicated with hepatitis and other lesions of the liver; and shall now shortly allude to the other position which M. Bonnet maintains—that inflammation of the parenchyma of the liver is almost always accompanied with inflammation of its peritoneal covering. It is rather strange that he has advanced this doctrine in so unqualified a manner as he has done, without being ready to support it by the testimony of numerous facts. Most unfortunately for M. B. the very contrary of his proposition appears to be nearer the truth: we strongly suspect that he is but indifferently well acquainted with the researches of one of the best modern pathologists. It is rare to meet with a French author who is at all conversant with English medical literature; but we might certainly have expected that he was so with the writings of his own countrymen. Now both Andral and Louis, the two great authorities on pathological subjects, have alluded to the rarity of hepatic peritonitis, compared at least with inflammation of other serous membranes, as the pleura. Morbid adhesions of the liver are by no means very frequently found on dissection; and often is that viscus itself altered, nay, even seriously disorganized, and yet we discover little change in its investing membrane. Mr. Annesley, in his recent very valuable work on the Diseases of India, thus expresses his opinions on this subject:—"We frequently observe in India the internal structure of the liver inflamed to the greatest possible extent, without any effusion of lymph from its surfaces, and the inflammation may go on to the production of several abscesses in both its lobes, or of one very large abscess in the right lobe only, without any decided marks of inflammation of the envelope of the organ, except some alterations of colour merely, which are usually occasioned by the state of the parts immediately underneath; nay, even abscesses of the liver may proceed to the utmost extent, and ultimately break into the abdominal cavity, without having induced inflammation of the serous surface where they point, and consequently without forming adhesions to the parts with which they are in immediate and close contact."

So much for the pathological information in M. Bonnet's work.

The description which he gives of the symptoms of hepatitis, acute and chronic, is tolerably good. En passant, we may remark that the alleged pain in the right shoulder, which used to be always enumerated as one of the most constant symptoms of diseased liver, is often quite absent, and the practitioner, therefore, is to be cautioned not to allow his diagnosis to be much influenced by this consideration.

The examination of the abdomen ought never, as a matter of course, to be omitted. When the abdominal parietes are thin, and the liver is at the same time considerably increased in volume, we find no difficulty in tracing its edge; but if the patient be fat, and the chest very capacious, it requires the greatest nicety of examination to obtain any satisfactory knowledge in this way. The tumefaction may be as great in the epigastrium, and even in the left hypochondrium, as in the right hypochondriac region; and the patient may experience as much uneasiness or pain, when we press on one of these parts, as on the other. If the disease be confined chiefly to the left lobe of the viscus, the right hypochondrium is occasionally quite free from any fulness, while the epigastrium and left hypochondrium only are distended and painful. Under these circumstances, the case may be mistaken for some tumor of the stomach, or even for disease of the spleen. Every experienced physician must have occasionally met with such cases. The liver attains sometimes such an enormous bulk—weighing 15, 20, 30 pounds and upwards—that it has been known not only to occupy the epigastric and two hypochondriac regions, but also to extend to, and even below, the umbilicus, reaching to the edge of the right os ilii.

Such cases are usually met with in hard-drinkers, or in patients who have suffered long and severely from attacks of intermittent fever. We need scarcely say, that it is almost next to impossible to form a very accurate diagnosis under these circumstances, unless the patient has been seen repeatedly during the progress of the enlargement. But in the less advanced form of the disease, the enlightened physician may generally succeed, although even then it often requires the nicest discrimination to do so. An hepatic tumor situated in the epigastric region may in general be distinguished from any tumor of the stomach itself, by its being more diffused and more moveable, and by its edge being more distinctly felt in the left than on the right side, where it almost always may be traced extending upwards under the cartilages of the ribs, and where therefore it is quite impossible to ascertain its boundaries. We need not allude at present to the assistance to be derived from the history of the case, and from the symptoms which particularly denote stomachic, and those which indicate hepatic derangement.

As to hepatic tumors simulating those of the spleen, we must remember, however improbable the statement may at first sight seem, that cases have occurred where the best physicians have erred in their diagnosis. In both, there is the same general diffused fulness of the side, the same dull pain, the same inability to lie on a particular side, the same predisposing and exciting causes, the same tendency to dropsical effusion into the abdomen, and so forth. Tumors however of the spleen are observed to be usually inclined from the left to the right side, and obliquely from below upwards, whereas the direction of those of the left lobe of the liver is more horizontal from the epigastrium towards the left hypochondrium. Icteric symptoms, too, are much less frequent in the one case than in the other.

In the preceding remarks, we have supposed that one or more lobes of the liver are enlarged in size, and that their misplacement has been caused by this enlargement. But it deserves to be noticed that this viscus may be felt not only in the epigastric, but also in the umbilical and left hypochondriac regions, although it has undergone no change in bulk. This may happen, when there is a copious effusion in the right cavity of the pleura; the diaphragm is thus forced down and the liver is made to protrude much lower than in health. M. Andral mentions a case in which a large encysted tumor had formed between the right kidney and the liver, and in which the latter viscus was fairly protruded into the left hypochondrium. The possibility of such sources of error should be known to every physician.

It occasionally happens that the gall-bladder is protruded so much downwards and outwards, that it forms a very distinct tumor at some part of the abdominal surface. Andral states that he has seen it either immediately below the cartilages of the right ribs, or lower down in the hypochondrium, or in the right iliac fossa, or lastly in the epigastrium. We can readily suppose that a tumor caused by the protrusion of the gall-bladder may be mistaken for an abscess, either of the liver itself or of the abdominal parietes. In the 1st vol. of the Dublin Hospital Reports, Mr. Todd has recorded a very curious case, where he committed this mistake, punctured the swelling, and the patient died of peritonitis on the following day. The facts are these. A young girl, after repeated attacks of fever, had been seized with excruciating pain in the abdomen, jaundice, &c. She was almost insensible when Mr. Todd first saw her. The epigastric and right hypochondriac regions were occupied with a tense swelling, at the most prominent point of which a sense of fluctuation was distinctly perceptible. A puncture being made, a thin fluid coloured with green bile flowed out; and when a canula was introduced into the wound, upwards of two quarts of a viscid bile were discharged. On dissection, the peritoneum was found inflamed and containing a quantity of serous and bilious fluid; the liver was quite healthy, the gall-bladder empty, but the hepatic and common ducts were so enormously distended, that they formed a sac in which there was still more than a quart of bile!

What adds greatly to the occasional difficulty of a correct diagnosis in hepatic disease, is the circumstance of its sometimes continuing for a length of time, and inducing the most extensive and most serious lesions of structure, without being indicated by any very prominent symptoms during life. Often is the liver found to be enlarged, hardened, tuberculated, or even to be in a state of suppuration, and yet the patient may not have once complained of pain or uneasiness in the side; the functions of the bowels may have been uniformly regular, and the skin have always been quite free from jaundiced colour. It certainly does appear very strange that the tissue of the liver may be seriously affected with diseased changes, while its secretion, the bile, may be little, if at all, changed from the healthy standard. But so it is: every pathologist knows the fact, and every work in general pathology records numerous instances to prove its truth. Andral mentions a case, where he found on dissection numerous abscesses, with redness and softening of the hepatic tissue around them, yet the patient never had pain in the region of the liver, nor had there ever been one sign of jaundice: he had died of acute pneumonia and gastritis. In another case, M. A. discovered an hepatic abscess associated with gangrene of the surrounding tissue, although there had been no symptom of liver-disease during life. But it is unnecessary to multiply examples. The great lesson to be drawn from all these considerations is the necessity of caution, we had almost said of *humility*, in judging of the nature and consequence of any disease, however inconsiderable it may seem to our imperfect faculties.

Those hasty men, who talk of physic as if it was one of the exact sciences, who profess to gauge and to measure diseases by the line and by the plummet of unerring description, and who make a boast of being able to propound certain "formulas," which will infallibly direct the practitioner in the cure of these, are little to be trusted. Very different is the conduct of the enlightened observer of Nature. Though quick in detecting disease, candid in expressing his opinion of its nature and its seat, and decided in his plans of treatment, he always avoids dogmatism of judgment, and rashness of action in his practice; knowing well that far more mischief is done by the over-officiousness, than by the cautious reserve of medical men. One of the most important, and certainly one of the most difficultly-acquired lessons in the healing art, is to know when to "do nothing." This is the secret of the "Médécine expectante" of some of the French physicians; and also of the foolish quackery, known in the present day under the title of homœopathism.* We cannot illustrate the value of these remarks better than by extracting the following most instructive remarks from Andral's Précis d'Anatomie Pathologique, on the extreme uncertainty of forming an accurate diagnosis of diseases of the liver.

"There is scarcely one of the alterations, to which the liver is subject, which has not been designated by the name of hepatitis. In my opinion, there is hardly one of them which may not be the result of an irritation, whose first effect was to induce an hyperæmia of the liver. On the other hand, I do not know an alteration of secretion, or of nutrition of the liver, not even a collection of pus in its parenchyma, that can be considered as necessarily arising from a previous state of irritation. I do not know one of which we can say, that its formation has been *necessarily* preceded by a hyperæmia."

The remaining page or two we shall devote to the consideration of one of the occasional effects of diseased liver—jaundice. There are some points in the history of this disease, which it is not easy to explain, and even in the present day, it will be found difficult in numerous instances to say, on what particular morbid state of the biliary apparatus it depends.

The symptoms and phenomena of the disease are occasionally variable and inconstant. In some rare cases, the yellow discoloration affects almost all the internal parts of the body, while the skin is nearly quite exempt from it. M. Ferrus mentions the case of a woman who died at the Salpetrière :—"Most of the solid parts of the body, with the exception of the skin, were found stained of a deep icteric dye; the subarachnoid tissue, and the adipose substance, in almost every part, were infiltrated with a saffron-coloured serosity. The liver was large and gorged with blood, but there was no obstacle to the passage of the bile from the gall-bladder into the bowels, as the gentlest pressure on the former caused the bile to flow freely along the common duct." Among other occasional anomalies of jaundice, we may mention that the discoloration of the surface may be limited to certain parts of the

* The exhibitions of some of our confrères in this metropolis, in reference to the absurdities of Hahneman's reveries, have of late been truly amusing. The "would-be," but "afraid-to-be" advocates of this nonsense most solemnly protest against being dubbed as homœopathists! Well may it be said of them—"they run with the hare and hold with the hounds." We fear that even the *innocent* hares will disown their most timid companions.—*Rev.*

body; as to the face, the chest, and even to one lateral half of the body. It is not easy to explain the occurrence of these irregularities; and indeed we cannot be much surprized at this, when we remember that the etiology of jaundice is very imperfectly understood. Hitherto it has been too customary to attribute every case of this malady either to disease of the liver, (which is then supposed to secrete an unhealthy bile, or a deficient quantity of it), or to an obstruction to the free passage of the bile from the gall-bladder into the duodenum, in consequence of gall-stones, the pressure of the adjoining viscera, spasm of the ducts, and so forth.

Now it is quite true that jaundice may, and that it does frequently arise in one or other of these manners; but it is equally true that, in some cases, the unprejudiced pathologist is puzzled to give a rational interpretation of its occurrence. We have already stated that jaundice is by no means an invariable concomitant of inflammation or other diseases of the liver. The skin indeed may assume a dull, yellowish, muddy hue; but this is very different from the saffron staining of genuine jaundice; and, on the other hand, every practical physician knows that the colour of the alvine evacuations is most irregular in this disease. Our author is quite correct, when he remarks: "les auteurs pretendent que dans l'ictère les dejections alvines sont pour l'ordinaire decolorées, grisatres, presque blanches; mais je crois pouvoir assurer qu'il est beaucoup plus commun qu'on ne pense de les voir conserver leur couleur naturelle." By attending to these circumstances, we are led to perceive how fallacious must be the doctrine, formerly prevalent, which referred every case of jaundice to an obstructed flow of bile from the gall-bladder into the intestines.

One of the most puzzling problems in the history of jaundice is, to account for its being caused by certain violent emotions of the mind, more especially by anger and deep grief, and also by severe bodily pain. It is not uncommon to find jaundice supervene on severe surgical operations, gunshot wounds, and strangulated hernia; and numerous examples of its occurring after fits of passion, of sudden alarm, and of overpowering affliction, are recorded by Morgagni, Lallemand, and Portal. Criminals, when sentence of condemnation has been passed upom them, have been known to become suddenly jaundiced. In all these cases, the disease has been regarded as purely nervous, and to be caused by a spasmodic affection of the gall-bladder or of its ducts. M. Bonnet is unwilling to admit this interpretation, and, ever pleased to mount his hobby-horse, he prefers to solve (?) the difficulty in the following way:—"Les accidens generaux qui accompagnent les grandes operations de chirurgie, les plaies d'armes-à-feu, &c. debutent toujours par la tube alimentaire, et l'on peut sans crainte avancer que l'irritation du parenchyme hepatique n'est jamais alors que consecutive à celle des voies digestives. C'est aussi par l'intermediaire d'une irritation gastro-intestinale que les vives affections de l'ame, les passions tristes ou gaies, determinent l'ictère." In this roundabout way, our author endeavours to explain the occurrence of jaundice-symptoms after injuries of the head, during pregnancy, and also in infants during the first week after birth. It is much more probable that the liver is directly and primarily affected in some of the cases alluded to above—upon sudden and violent emotions of the mind, severe and protracted bodily pain, injuries of the head, &c.—than that the hepatic affection is secondary and consecutive upon a gastro-intestinal irritation. There seems to be no necessity for this additional link in the diseased action, and we therefore think that it is wiser and better to affirm simply, with M. Andral, "que le cerveau re-agit directement en quelques circonstances sur le plexus hepatique."

But in saying this, we are very far from denying the frequent connexion of jaundice with a subacute inflammation of the bowels, more especially of the duodenum. In truth, this complication is much more common than has been generally supposed, at least in this country. If the patient feels pain or uneasiness on pressure of the epigastrium, if he is thirsty and feverish, if the tongue be dry, and red at the edges, if the skin be dry and harsh to the feel, and, above all, if there is a marked depression of the strength and powers of the system, then has the physician reason to suspect, that the case is not one of merely obstructed flow of the bile, but rather that the jaundice is only one symptom of a gastro-enteric inflammation.

The sympathy between the diseased mucous membrane and the liver and gall-bladder is quite sufficient to induce a most complete jaundice, although there may be no mechanical obstruction of the ducts, and the alvine evacuations have, during the life of the patient, exhibited no apparent deficiency of bilious admixture. The jaundice which sometimes follows the drinking of cold fluids, or the exposure to cold weather when the body is heated, and that also which is consequent upon a debauch in the luxuries of the table, is very generally associated with an inflamed state of the gastro-duodenal surface. But a form of the disease, which is more common than either of these now mentioned, and more important, too, from its being very obscure and very dangerous, is the jaundice which is associated with and constitutes an essential feature of certain fevers. Such are the bilious fevers of hot climates, and such, also, are some epidemics which we occasionally meet with in this country.

In these pyrexiæ, there can be little doubt that the gastro-enterite precedes the coming on of the hepatic symptoms. We are the more anxious to direct, in an especial manner, the attention of our readers to this circumstance, because, if the assertion be true, the indiscriminate use of active acrid purgatives in fever—a practice very prevalent among British physicians—ought to be reprobated.

Our limits prevent us from examining this important subject at greater length. What we have chiefly aimed at, in the preceding pages, is to establish the following propositions—that the diseases of the liver, in order to be rightly understood, must be studied not apart and by themselves, but in connexion with diseases of other parts of the body—that some are primary and idiopathic, such as those which occur in tropical climates, after intermittent fevers, after injuries of the right hypochondrium and even of distant parts, as of the head; and to these we may add the morbid affections which follow upon violent mental emotions, and protracted bodily suffering—that many are secondary, and are symptomatic of, or at least are complicated with, a subacute inflammation of the mucous membrane of the stomach and duodenum—that diseases of the liver may exist long, and may induce extensive alteration in the hepatic tissue, without being attended by any characteristic symptoms during the life of the patient—that the tissue of the liver may be seriously diseased, and yet the bile may be secreted normally in point of quality and properties—that therefore jaundice is not a necessary symptom or accompaniment of morbid liver—that this disease, jaundice, often occurs, when the gall-bladder is found after death to be full of healthy bile, and there is no mechanical obstruction to the flow of this fluid from the gall-bladder into the intestines—and lastly, that many cases of jaundice, especially that form of it which occurs in bilious fevers, depend upon an inflammation of the gastro-duodenal surface.

The attentive consideration of these propositions leads to some important practical conclusions. It shews us the impropriety of grouping together a multitude of cases, which may have only a few symptoms in common, under one general denomination of diseases of the liver, bilious affections—an error which has been much more common among British than among continental pathologists—without regard to their history, their causes, symptoms and complications. We have neither space nor leisure to discuss the treatment of these various and varied diseases. Moreover the labour would be quite unnecessary; for in general it is not difficult to apply the appropriate remedies, when we have once formed an accurate diagnosis of a case. There are, however, one or two topics to which we shall very briefly allude. Every one knows that mercury, in some form or another, has been long deemed the grand specific in diseases of the liver. That this metal unquestionably possesses wonderful effects in checking the progress of acute hepatitis cannot be denied by any physician. All tropical writers agree on this score, and the almost universal belief is, that if salivation can be once established in a case, the inflammatory process is almost instantly arrested. It is a curious fact in the history of therapeutics, that this most potent subduer of inflammation, mercury, should have been in common use against one or two of the phlegmasiæ—hepatitis and iritis—for a great length of time, before it was tried in other diseases of the same class. It has been only of late years that the great efficacy of long and repeated doses of calomel, in inflammations of the serous and fibrous tissues, in rheumatism, &c. has been fairly established. The error is to be explained thus.

It was long believed that mercury had some *peculiar* and specific influence on the liver; and this doctrine may be, to a certain extent, quite correct. But that its efficacy in cases of hepatitis is to be accounted for just in the same manner as its equal efficacy in many other inflammations—by inducing perhaps an opposing and counter-acting action in the capillary vessels, an action which is incompatible with the inflammatory one*—will not, we suppose, be denied by any one in the present day.

It is an unfavourable symptom in inflammatory and febrile diseases, when the system resists the mercurial action. This circumstance, therefore, taken in connexion with other symptoms, such as abatement of the pain, shiverings, &c., may suggest to the practitioner some conjecture of the changes that are about to take place. Mr. Annesley remarks, "that there can be no doubt that the system will not be brought under the full operation of mercury, or that ptyalism will not follow on the most energetic employment of this substance, when abscess exists, although a slight tenderness of the gums will be produced by it."

---

* The study of the modus operandi of mercury in arresting inflammatory disease may possibly throw some light on the state of the capillaries in an inflamed part—the proximate cause of inflammation. The little knowledge that we have of this subject tends rather to favour the doctrine of Vacca, that there is a stasis, or stagnation of the blood in the extreme vessels.—*Rev.*

In the more chronic affections of the liver, some one of the preparations of mercury is very generally exhibited in conjuuction with other medicines, in the view of promoting the flow of the bile. The practice is perfectly safe and proper in some cases; but the indiscriminate use of this mineral is certainly to be deprecated. It is even doubtful, whether mercury is more decidedly a cholagogue than many others of the class of purgatives. The alkalis, more especially the subcarbonas and the liquor potassæ in union with the decoct. aloes compos. of our Pharmacopœia, or with some of the preparations of colchicum, will be found admirable remedies. If the flow of the urine be solicited, at the same time that the action of the bowels is stimulated, the relief to the patient will be more decided. The sal. polychrestus of the older pharmaceutists, the sulphas. potassæ of our pharmacopœia, and the Rochelle salts, the tartras sodæ, in combination with a few grains of jalap, or of rhubarb, are most useful for this purpose. We need not again urge on our readers' attention the importance of ascertaining in all cases, whether there be any irritation, or inflammatory action of the gastro-enteric surface. When we have reason to suspect the presence of such a complication, the repeated application first of leeches, and then of rubefacients or blisters to the epigastrium, the internal use of refrigerants, such as small doses of nitre, of borax, and the sucking of pieces of ice, the employment of enemata, and then of the mildest aperients, such as castor oil, by the mouth, along with a most rigid " diete," will in almost every case remove the insidious enemy. The use of the nitro-muriatic baths has, of late years, been perhaps rather too much neglected in the treatment of chronic diseases of the liver.

Here we must stop. We have hitherto given no opinion of the merits of M. Bonnet's work. It obtained the gold medal from the Societé Medicale d'Emulation of Paris. Like most prize essays, it is of very mediocre merit.

